# A Volatile Metabolomics Perspective: Interplay Between Indigenous Lactic Acid Bacteria and Aroma Development in Ripening Raw-Milk Cheese

**DOI:** 10.3390/foods15142411

**Published:** 2026-07-08

**Authors:** Milena Alicja Stachelska, Mariusz Banach, Piotr Karpiński, Bartosz Kruszewski

**Affiliations:** 1 Faculty of Health Sciences, University of Lomza, Akademicka 14, 18-400 Łomża, Poland; mbanach@al.edu.pl (M.B.); pkarpinski@al.edu.pl (P.K.); 2 Department of Food Technology and Assessment, Institute of Food Sciences, Warsaw University of Life Sciences—SGGW, Nowoursynowska 159 C, 02-776 Warsaw, Poland

**Keywords:** artisanal cheese, volatile organic compounds (VOCs), LAB growth, microbial succession, aroma development, HS-SPME/GC-MS, indigenous microflora

## Abstract

Artisanal raw-milk cheese represents a complex biochemical ecosystem where the indigenous microbiota acts as the primary driver of the volatile profile. This study utilizes an innovative synchronized biological relay model to decipher the mechanistic interplay between the successional dynamics of indigenous lactic acid bacteria (LAB) and the temporal evolution of the volatile metabolome over a 10-week maturation period of an artisanal cow-milk cheese. Utilizing a culture-dependent approach focused on the quantitative enumeration of broad morpho-physiological groups—without species-level identification—integrated with HS-SPME/GC-MS, we characterized the precise shifts from early-stage lactic cocci to dominant rod-shaped lactobacilli. Initial populations at Week 0 consisted of 8.2 log CFU/g of cocci and 4.1 log CFU/g of rod-shaped LAB. Lactic cocci peaked at Week 2 (8.5 log CFU/g) before undergoing mass autolysis down to 7.1 log CFU/g by Week 4, releasing intracellular enzymes that catalyzed a 900% surge in total esters and a 215% increase in volatile alcohols. Concurrently, rod-shaped LAB proliferated to a maximum of 8.6 log CFU/g at Week 6, directly correlating with a 125% increase in total carboxylic acids, prominently driven by a 750% accumulation of hexanoic acid. The late-phase maturation (Weeks 8–10) established a technological equilibrium: volatile sulfur compounds collapsed by over 90% within the first two weeks, initial transient lactones were replaced by a 1200% late-stage increase in dodecalactone, and matrix-sequestered dietary terpenes were liberated via an 8-fold (700%) increase at Week 8. This study introduces an innovative, statistically validated volatilomic framework that equips the dairy sector with an advanced metabolomic tool for rigorous product authentication and targeted flavor optimization, thereby establishing a scientific baseline for the reproducible production of premium, organoleptically superior artisanal cheeses.

## 1. Introduction

The manufacture of traditional cheeses from non-pasteurized bovine milk constitutes a multifaceted biochemical transformation, where the intrinsic microbial ecology of the raw material serves as the primary driver of the final sensory profile [1]. Unlike standardized industrial productions that often rely on thermal treatment and commercial starter cultures, artisanal raw-milk cheeses depend on a diverse autochthonous microbiota [2]. These native populations, primarily lactic acid bacteria (LAB), survive the initial stages of curd formation and drive extensive enzymatic reactions throughout the maturation phase. This natural microbial reservoir is fundamental not only for preserving cultural heritage but also for developing the distinct organoleptic characteristics that confer uniqueness to traditional dairy products [3,4]. In this specific ecological niche, raw bovine milk functions as a complex substrate where the initial microbial load acts as a biological catalyst, transforming primary nutrients into a wide array of aromatic metabolites over a long-time ripening period [5].

The maturation of raw cow-milk cheese is fundamentally maintained by the dynamic succession of the indigenous lactic acid bacteria (LAB) naturally present in the unpasteurized milk substrate [6]. This biological process begins with the proliferation of autochthonous lactic cocci, which facilitate the initial fermentation stages and establish the necessary chemical environment for subsequent ripening phases. As the physical and chemical properties of the maturing curd evolve—specifically through the gradual reduction in moisture and the escalation in acidity—a distinct successional transition occurs, leading to the dominance of resilient, rod-shaped lactobacilli [7,8,9,10]. These indigenous non-starter lactic acid bacteria (NSLAB) are uniquely adapted to the rigorous conditions of the aging matrix, allowing them to remain metabolically active throughout the ripening period. Consequently, these populations serve as the primary biological drivers that catalyze the diverse secondary metabolic pathways required for the development of a complex and sophisticated aroma profile [11,12].

Flavor development in raw-milk cheese is primarily driven by these microbial shifts, which trigger a cascade of biochemical reactions including the fermentation of citrate and residual sugars, proteolysis, and lipolysis. These pathways result in the production of a sophisticated matrix of volatile organic compounds (VOCs) that serve as the primary contributors to the cheese’s unique aroma [13,14]. To achieve a high-resolution mapping of these ripening dynamics, contemporary research emphasizes the comprehensive characterization of a broad spectrum of volatile chemical classes. The biosynthesis of these diverse volatile organic compounds is fundamentally driven by the metabolic activity of the milk’s indigenous microbiota. The scientific literature indicates that carboxylic acids and esters are recognized as primary products of lipolytic and esterification pathways, contributing the characteristic piquant and fruity aromatic notes to the mature curd [9,15,16,17]. Furthermore, volatile alcohols and terpenes often function as indicators of amino acid catabolism or dietary carry-over from the raw milk, thereby imparting delicate floral and herbaceous nuances to the sensory profile. Concurrently, sulfur-containing compounds and lactones serve as transient odorants and intermediate metabolites that define the depth, complexity, and overall organoleptic distinctiveness of the final cheese aroma [14,18,19].

Accurate monitoring of these chemical and microbiological changes traditionally relied on culture-dependent enumeration of selective bacterial cohorts integrated with gas chromatography-mass spectrometry to profile low-concentration odorants within the mature curd. However, contemporary dairy science has experienced a profound paradigm shift toward high-throughput, culture-independent innovations that bridge the gap between metagenomic codes and volatile signatures. Recent advancements emphasize that multi-omics approaches are currently essential to fully clarify the complex functional interactions within wild microbial communities, linking taxonomic shifts with real-time flavor and quality development [20,21]. The implementation of next-generation sequencing (NGS) platforms, particularly shotgun metagenomics, has made great strides by transitioning from purely descriptive surveys to high-resolution functional mapping of raw-milk cheese ecosystems, thereby unraveling the exact biochemical mechanisms governing lipolysis and caseinolitic degradation [22]. This integrated metataxonomic and volatilomic profiling allows researchers to track open-fermentation successions from manufacture to late-stage ripening, explicitly connecting the proliferation of unculturable starter and non-starter cohorts with the accumulation of short-chain fatty acids, alcohols, and esters [23,24]. Furthermore, combining advanced shotgun metagenomic assemblies with phenotypic culture traits confirms that the genetic potential of indigenous lactic acid bacteria directly dictates the generation of specific volatile organic compound (VOC) waves in raw-milk artisanal systems [25]. Yet, while high-throughput sequencing creates comprehensive qualitative blueprints of the cheese fermentome, a critical analytical gap persists regarding the absolute, real-time kinetic quantification of viable microbial transitions under evolving physicochemical gradients. Consequently, bridging these contemporary multi-omics and metagenomic insights with a robust, chronologically synchronized quantitative tracking framework remains a mandatory prerequisite to transform descriptive volatolomic fingerprints into reproducible tools for product authentication and quality control in the traditional dairy sector [17].

Traditional artisanal cheesemaking utilizing unpasteurized milk suffers from profound technological vulnerability due to the inherent unpredictability of the raw material’s native microflora, leading to severe batch-to-batch flavor inconsistency. While the dairy industry frequently mitigates this volatility by adopting commercial starter cultures, these conventional interventions fundamentally compromise the multi-layered sensory complexity and geographical typicity that define premium raw-milk products. Consequently, producers face a critical technological dilemma, as they lack reliable mechanisms to standardize ripening outcomes without sacrificing the authentic organoleptic traits driven by native lactic acid bacteria (LAB). Concurrently, existing volatilomic and metabolomic research remains heavily constrained by retrospective, purely descriptive mappings of cheese aroma, which fail to offer actionable biochemical thresholds. Current analytical paradigms fail to establish a precise, quantitative link between the chronological phases of indigenous microbial succession—such as the exact timing of lactic cocci autolysis—and specific volatile synthesis waves. This knowledge deficit prevents the development of targeted quality control systems capable of managing the biochemical transition from early acid formation to advanced flavor maturation in non-pasteurized matrices. Therefore, a clear technological gap persists for a synchronized, quantitative framework capable of transforming raw metabolomic profiles into an actionable tool for flavor optimization and product authentication. The aim of this study is to explore the 10-week maturation process of artisanal raw cow-milk cheese using a metabolomics-driven approach to correlate the successional growth of native microflora with the biosynthesis of volatile organic compounds (VOCs). Specifically, this research seeks to correlate the microbial succession—defined by the transition from initial cocci populations to dominant rod-shaped lactobacilli—with the specific volatile markers. A comprehensive metabolomic profiling via HS-SPME/GC-MS was utilized to track the biosynthesis of diverse VOCs, including carboxylic acids, esters, alcohols, lactones, terpenes, and sulfur compounds. Particular attention is given to carboxylic acids and esters, as primary products of lipolytic and esterification pathways, as well as alcohols and terpenes, which serve as indicators of amino acid catabolism or dietary carry-over from the raw milk. Finally, sulfur-containing compounds and lactones are examined for their roles as potent odorants and intermediate metabolites. Elucidating these correlations is intended to establish a scientific basis for quality control and the chemical authentication of raw-milk products based on their signature volatile profiles, highlighting the role of native flora in defining the unique volatile matrix of traditional artisanal cheeses.

## 2. Materials and Methods

### 2.1. Cheese Production

Artisanal cheese was manufactured using raw, unpasteurized cow’s milk obtained from a single morning milking. Milk, characterized by a fat content of 4.8% (*w*/*v*), a protein content of 3.6% (*w*/*v*), and a lactose content of 4.7% (*w*/*v*), was sourced from a selected organic farm in the Podlasie region of Poland. No commercial starter cultures or external ripening microorganisms were added, ensuring that the fermentation and subsequent aroma development were entirely governed by the milk’s indigenous microbiota.

Prior to the cheesemaking process, the microbiological quality of the raw milk was assessed. Total mesophilic aerobic bacteria were enumerated using Plate Count Agar (PCA) incubated at 30 °C for 72 h, in accordance with the ISO 4833-1:2013 standard [26]. All analyses were performed in triplicate. The raw milk exhibited high microbiological quality, with a total aerobic bacterial count of 4.2 × 10^4^ CFU/mL, ensuring a controlled starting point for the indigenous fermentation process.

The production process was initiated by heating the raw milk to a temperature of 32 °C. Upon reaching this temperature, calf rennet was added to induce coagulation, which occurred within a thirty-minute period. The resulting curd was cut into cubes approximately 5 cm thick and then gradually reheated to 42 °C over a period of 25 min (at a heating rate of approximately 0.4 °C/min) under continuous, gentle stirring to facilitate uniform whey expulsion and prevent case hardening of the curd grains. After the heating stage, the cheese mass was transferred to perforated cylindrical plastic molds (diameter 15 cm, height 10 cm) for drainage and acidification at 22 °C for 48 h. No external mechanical pressure was applied. Instead, whey expulsion was achieved entirely through self-pressing via gravitational drainage under the curd’s own weight. To ensure uniform moisture distribution, symmetrical acidification, and a homogeneous structural matrix, the cheese wheels were systematically inverted according to a strict schedule: every 30 min during the first 2 h, followed by inversions at 6, 12, and 24 h, and twice daily for the remainder of the acidification phase. Following the acidification phase, the shaped cylindrical cheese wheels were salted by immersion in a brine solution. The brine was prepared with a concentration of 15% (*w*/*v*) NaCl, maintained at a constant temperature of 12 ± 1 °C to align with the subsequent maturation climate, and adjusted to a pH of 5.25 using food-grade lactic acid (85% *w*/*w*). This pH adjustment was critically performed to establish an ionic and thermodynamic equilibrium between the solution and the acidified curd, thereby preventing the migration of structural calcium (Ca^2+^) ions from the cheese matrix into the brine, which would otherwise destabilize the casein network and induce rind softening defects. The cheeses remained in the brine for a duration of 48 h to ensure adequate salt penetration. Finally, the cheeses were transferred to a controlled maturation chamber (Climacell 111, BMT Medical Technology, Brno, Czech Republic) for the ripening process. The ripening phase lasted for 10 weeks (70 days), during which the chamber was maintained at a constant temperature of 12 °C and a relative air humidity of 80–85%.

The cheesemaking experiment was conducted in two independent batches. In each batch, 9 cheese wheels were produced, yielding a total of 18 wheels for the entire study.

### 2.2. Cheese Sampling

The maturation process was monitored bi-weekly across six sampling points: Week 0 (immediately after production), Week 2, Week 4, Week 6, Week 8, and Week 10. At each sampling interval, three cheese wheels were randomly selected and removed from the maturation chamber for analysis. From each selected wheel, subsamples were extracted aseptically from three different locations (core, middle, and sub-rind areas). These subsamples were then combined to obtain a single representative composite sample for each investigated cheese wheel. This sampling strategy ensured that the analytical results accounted for both the successional dynamics of the native microflora and the spatial heterogeneity of the cheese matrix.

For microbiological analysis, 10 g was weighed from each composite sample in accordance with the ISO 15214:1998 standard [27]. The samples were obtained using a sterile stainless-steel borer to reach the interior zones, immediately transferred into sterile bags, and transported under refrigerated conditions (4 °C) to the laboratory for the enumeration of LAB.

For the characterization of the volatile profile, sampling was conducted simultaneously using the same aseptic technique. The combined inner cheese mass (excluding the rind) of each wheel was grated to ensure homogeneity prior to weighing.

### 2.3. Microbiological Procedures for Indigenous Microflora Assessment

The enumeration of lactic acid bacteria (LAB) populations, specifically lactic cocci and rod-shaped lactobacilli, was conducted at bi-weekly intervals, Week 0–Week 10. A cheese sample was aseptically weighed and homogenized in 90 mL of sterile 2% (*w*/*v*) sodium citrate solution to prepare the initial dilution. Subsequent serial decimal dilutions were performed using sterile physiological saline (0.85% NaCl).

#### 2.3.1. Enumeration of Lactic Cocci

Lactic cocci were quantified using M-17 agar acc. to Terzaghi LAB-AGAR™ (BioMaxima S.A., Lublin, Poland). The medium was prepared at a concentration of 55 g/L in distilled water and sterilized in an autoclave at 121 °C for 15 min. M-17 agar is specifically designed for the isolation of lactic streptococci from dairy products and utilizes lactose (5.0 g/L) as the primary energy source. The medium features a high buffering capacity provided by sodium glycerophosphate (19.0 g/L), which maintains the final pH at 7.2 ± 0.2 at 25 °C. Growth was stimulated by the addition of ascorbic acid (0.5 g/L), and essential minerals were provided via magnesium sulfate. Inoculated plates were incubated in a laboratory incubator (CLW 53, POL-EKO, Wodzisław Śląski, Poland) at a temperature of 30 °C for 48 h.

#### 2.3.2. Enumeration of Lactobacilli Rods

The population of rod-shaped lactobacilli was determined using MRS LAB-AGAR™ pH 5.7 (BioMaxima S.A., Lublin, Poland). This medium was prepared by dissolving 70 g of the dehydrated product in 1 L of distilled water, followed by sterilization in an autoclave at 121 °C for 15 min. The medium is formulated in accordance with the ISO 15214:1998 standard [27] and utilizes a combination of enzymatic casein hydrolysate, yeast extract, and meat extract to provide nitrogen and carbon sources. Selective inhibition of competing Gram-negative bacteria and fungi was achieved through the inclusion of ammonium citrate (2.0 g/L) and sodium acetate (5.0 g/L). Tween 80 (1.08 g/L) was incorporated as a source of oleic acid esters to support the specific fatty acid requirements of *Lactobacillus* metabolism. Inoculated plates were incubated under microaerophilic conditions in an atmosphere enriched with 5% CO_2_ to optimize the growth of these species. The incubation was carried out in a laboratory incubator (CLW 53, POL-EKO, Wodzisław Śląski, Poland) at a temperature of 30 °C for 72 h. Following incubation, white, round colonies with smooth edges were counted.

### 2.4. Physicochemical Characterization

Standardized physicochemical analyses were performed in triplicate at each bi-weekly maturation interval (Weeks 0, 2, 4, 6, 8, and 10). Active acidity (pH) was determined potentiometrically using a digital pH meter (Edge HI2002, Hanna Instruments, Woonsocket, RI, USA) equipped with a solid-state glass penetration electrode calibrated with certified IUPAC buffer solutions at pH 4.01 and 7.01. Water activity (a_w_) was measured at a controlled temperature of 25 ± 0.5 °C utilizing a chilled-mirror dew point water activity meter (AquaLab 4TE, METER Group, Pullman, WA, USA). Total moisture content was quantified gravimetrically via hot-air oven drying at 102 ± 2 °C until a constant weight was attained in accordance with the ISO 5534:2004 standard [28]. Sodium chloride (NaCl %) content was determined using the classical Volhard argentometric method following titration with silver nitrate (AgNO_3_) and ammonium thiocyanate (NH_4_SCN) according to ISO 5943:2006 protocols [29]. Total fat content was quantified using the standard Van Gulik acid butyrometric method tailored for hard and semi-hard cheese matrices in compliance with ISO 3433:2008 standards [30].

### 2.5. HS-SPME/GC–MS Analysis

Portions of approximately 1 g were weighed for VOCs profiling analysis and placed directly into 20 mL clear glass headspace vials. Each vial was hermetically sealed with a PTFE/silicone septum and an aluminum crimp cap for subsequent HS-SPME/GC-MS analysis. Volatiles were collected from the headspace of the sample using a DVB/CAR/PDMS SPME arrow fiber (Shimadzu, Kyoto, Japan) after a 15-min incubation at 90 °C. Desorption was performed in the injector at 250 °C for 2 min [16]. Analysis was conducted on a Shimadzu Nexis GC-2030 GC/MS coupled with an AOC-6000 autosampler and a ZB-5MSi column (Phenomenex, Torrance, CA, USA) with a length of 30 m, an internal diameter of 0.25 mm, and a film thickness of 0.25 µm. The oven program started at 35 °C (held for 5 min), then ramped to 250 °C at a rate of 8 °C/min. Helium served as the carrier gas at a flow rate of 1.5 mL/min. Mass spectrometry was conducted in full-scan mode, covering a range of 40 to 500 *m*/*z* [31]. Compounds were identified using the NIST 14 library, MassBank, and the retention indices of n-alkane standards analyzed under the same conditions as the samples. To ensure the technical reproducibility of the volatile organic compound (VOC) measurements, each composite sample was analyzed in triplicate.

### 2.6. Statistical Analysis

All experimental analyses, including microbiological enumerations and HS-SPME/GC-MS measurements, were performed in triplicate. All results were expressed as the mean ± standard deviation (SD). To evaluate the impact of the ripening period (T0 to T5) on the concentration of volatile organic compounds (VOCs) and the population dynamics of LAB, a one-way ANOVA was conducted. Significant differences between the means were determined using Tukey’s post hoc test, with a significance level set at *p* < 0.05. To visualize the progress of volatiles profile formation during cheese ripening, a principal component analysis (PCA) was performed on the overall HS-SPME/GC-MS dataset. Statistical analyses were executed using the SPSS software package (Version 26.0, IBM Corp., Armonk, NY, USA).

## 3. Results and Discussion

### 3.1. Interplay of Physicochemical Dynamics and Indigenous Lactic Acid Bacteria Succession

The structural and organoleptic evolution of artisanal raw-milk cheese is fundamentally governed by a dynamic thermodynamic matrix, wherein core physicochemical parameters act as primary regulatory filters rather than secondary environmental factors. The chronological progression of these parameters across the 10-week maturation period is structured in Table 1.

**Figure 1:**
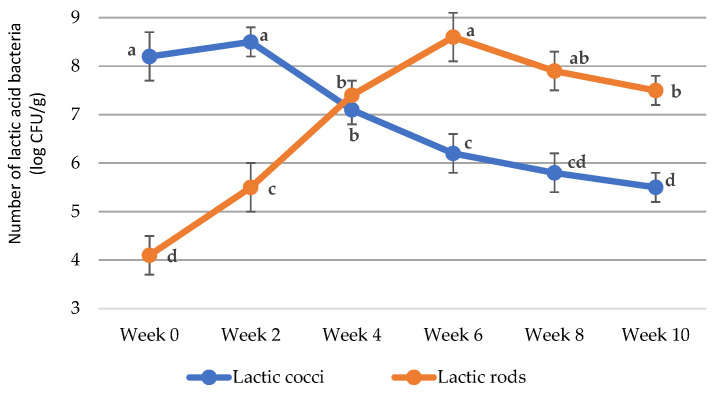
Changes in the counts of lactic acid cocci and lactic acid rods during the 10-week cheese ripening process. Values marked with different letters are significantly different (*p* < 0.05) and concern changes in the number of specific LAB during the time of ripening.

The structural parameters established at Week 0, characterized by an elevated moisture content of 44.21 ± 0.52% and a water activity (a_w_) of 0.976 ± 0.002, combined with an initial pH of 5.26 ± 0.03, established a highly supportive, nutrient-dense habitat for pioneering microorganisms. This specific physicochemical baseline directly shaped the initial successional dynamics of the native lactic acid bacteria (LAB) populations illustrated in Figure 1.

The commencement of the maturation process was defined by a profound numeric disparity between the two key non-starter lactic acid bacteria (NSLAB) cohorts, showing a clear predominance of indigenous lactic acid cocci at 8.2 log CFU/g against a substantially restricted population of rod-shaped LAB anchored at 4.1 log CFU/g. As demonstrated by Bettera et al. [32], this baseline microbiological configuration is highly representative of traditional unpasteurized dairy matrices, where raw milk serves as the primary biological reservoir for foundational *Lactococcus* and *Streptococcus* species that initiate the early cheese structure. Supported by the initial high moisture and optimal water activity, these pioneering lactic cocci rapidly fermented residual lactose during the first 14 days, driving a downturn in pH to 5.14 ± 0.02 and attaining their maximum population density of 8.5 log CFU/g at Week 2. According to Banks and Williams [33], the proliferation of this indigenous cocci flora critically regulates the rate of early-stage acidification, thereby directly influencing syneresis kinetics, moisture drainage, and the subsequent activation thresholds of residual rennet enzymes.

However, as chronological ripening advanced toward Week 4, continuous water evaporation within the controlled maturation chamber triggered a steady contraction of the moisture network down to 38.12 ± 0.39%, forcing a progressive concentration of sodium chloride to a distinct threshold of 1.88 ± 0.04%. This progressive moisture loss, paired with maximum active acidity at a pH minimum of 5.02 ± 0.04, imposed intense osmotic and thermodynamic stress upon the cell walls of the lactic cocci. This challenging micro-environment functioned as the definitive biological trigger for a severe successional transition between the second and sixth weeks of ripening, during which the cocci population experienced a sharp, continuous collapse, decreasing from its peak to 6.2 log CFU/g, marking a total reduction of 2.3 log units. As elucidated by Anastasiou et al. [20], this mass bacterial autolysis serves as a major catalytic turning point, as the physical degradation of the cocci cell membranes results in the systematic liberation of intracellular enzymes—including peptidases, esterases, and deaminases—which were previously sequestered within the cellular boundary. Furthermore, Ruvalcaba-Gómez et al. [34] established that the release of these intracellular enzymes into the core curd matrix profoundly accelerates flavor development by catalyzing complex esterification reactions between free fatty acids and volatile alcohols, thereby establishing the foundational aroma complexity of the product.

Conversely, this distinct modification of the physicochemical landscape prepared the ecological niche for the successional dominance of the more resilient, rod-shaped NSLAB. Surpassing the cocci counts at approximately Week 4, the rod-shaped LAB population adjusted effectively to the acid-induced proton gradients and concentrated salt levels, expanding exponentially to attain its absolute maximum abundance of 8.6 log CFU/g at Week 6 under a stable pH of 5.06 ± 0.03 and a restricted water activity of 0.948 ± 0.002. To provide a rigorous theoretical foundation for these findings, the community transitions observed herein can be evaluated through the classical secondary microflora model articulated by Beresford et al. [35]. In the general ecological succession literature, pioneering cohorts modify the habitat’s constraints, thereby facilitating the colonization of subsequent, better-adapted species. The framework of Beresford et al. illustrates how secondary rod-shaped lactobacilli uniquely colonize the post-fermentation matrix by expanding under adverse conditions and utilizing alternative energy substrates, such as citrates, lactates, and the exact amino acids made available following the autolysis of the primary flora.

Our proposed synchronized biological relay model builds upon this principle but directly links these demographic milestones to specific real-time volatolomic pulses. The cellular death of the early cocci inherited an active enzymatic framework that directly fueled the secondary metabolic vigor of the lactobacilli rods, whose advanced lipolytic and proteolytic activities, driven at the Week 6 population peak, catalyzed the maximum accumulation of core carboxylic acids and late-phase aroma refinement.

Following this maximum, the concluding phase of maturation (Weeks 8–10) established a natural state of technological equilibrium. Both microbial groups faced a gradual growth inhibition, with populations of rods and cocci stabilizing at 7.5 log CFU/g and 5.5 log CFU/g, respectively, by the end of the 70-day cycle. As noted by Mureşan et al. [36], this reduction in viable lactic acid bacteria counts toward the late stage of aging is fundamentally dictated by the severe depletion of fermentable carbohydrate substrates and the localized accumulation of toxic metabolic byproducts, which function as natural autoinhibitors. Concurrently, advanced proteolysis induced a slight buffering effect that raised the final pH to 5.22 ± 0.03, while moisture reduction down to 34.25 ± 0.28% stabilized water activity at a safe equilibrium of 0.941 ± 0.001. Simultaneously, fat content rose on a total weight basis from an initial 28.45 ± 0.38% to a final concentration of 34.82 ± 0.26%, representing a passive concentration effect induced by the global 22.5% reduction in total moisture content over the 70-day period. This progressive stabilization confirms that the artisanal raw-milk cheese successfully achieved optimal technological and organoleptic maturity while safely maintaining its distinct, pasture-derived volatile identity.

### 3.2. General Characterization and Evolution of Volatile Organic Compounds (VOCs)

The HS-SPME/GC-MS analysis of cheese samples at various stages of ripening allowed for the identification of numerous volatile organic compounds (VOCs) belonging to seven distinct chemical classes: alcohols, esters, carboxylic acids, lactones, terpenes, and sulfur compounds.

The total volatile abundance showed a progressive increase during the initial weeks, reaching a peak for carboxylic acids around Week 6, followed by a stabilization phase toward the end of the process (Weeks 8–10). This evolution reflects the intense metabolic interplay driven by the indigenous microflora, particularly through biochemical pathways such as lipolysis and proteolysis. These processes generate essential precursor molecules that contribute to the development of the complex aroma profile and sensory depth as the cheese reaches technological maturity [37].

**Figure 2:**
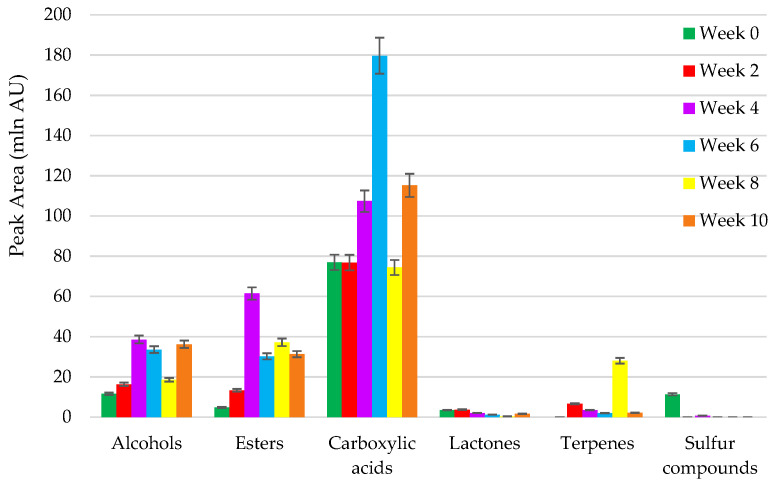
Successive changes in the profile of volatile organic compound (VOC) classes during the 10-week cheese ripening process.

**Figure 3:**
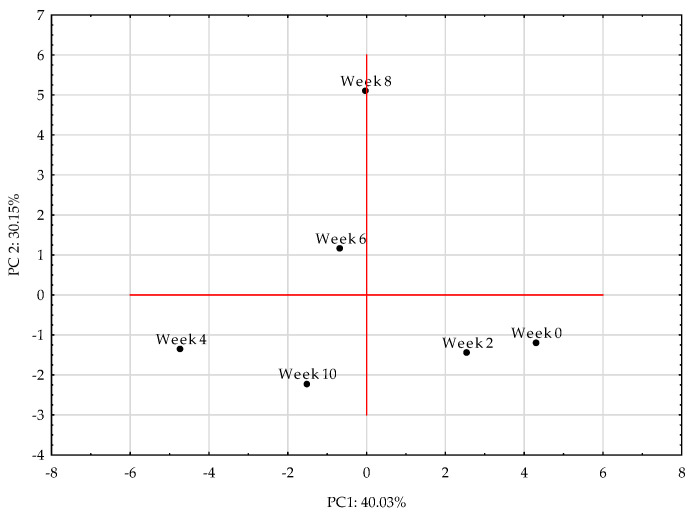
Principal component analysis (PCA) results. Score plot, PC1 versus PC2 of all samples taken during the 10-week cheese ripening process.

The results presented in Figure 2 constitute a comparative trend analysis, illustrating the dynamic formation, accumulation, and subsequent decline of specific volatile metabolites across the ripening stages. These chemical shifts show a strict correlation with the proliferative and inhibitory dynamics of the indigenous microflora originating from the raw cow’s milk. Specifically, the evolution of the VOC profile is directly driven by the metabolic activity of lactic acid cocci and rods, whose succession defines the stages of maturation. The dynamics in the VOC profile of cheese are shown in Figure 3.

**Figure 4:**
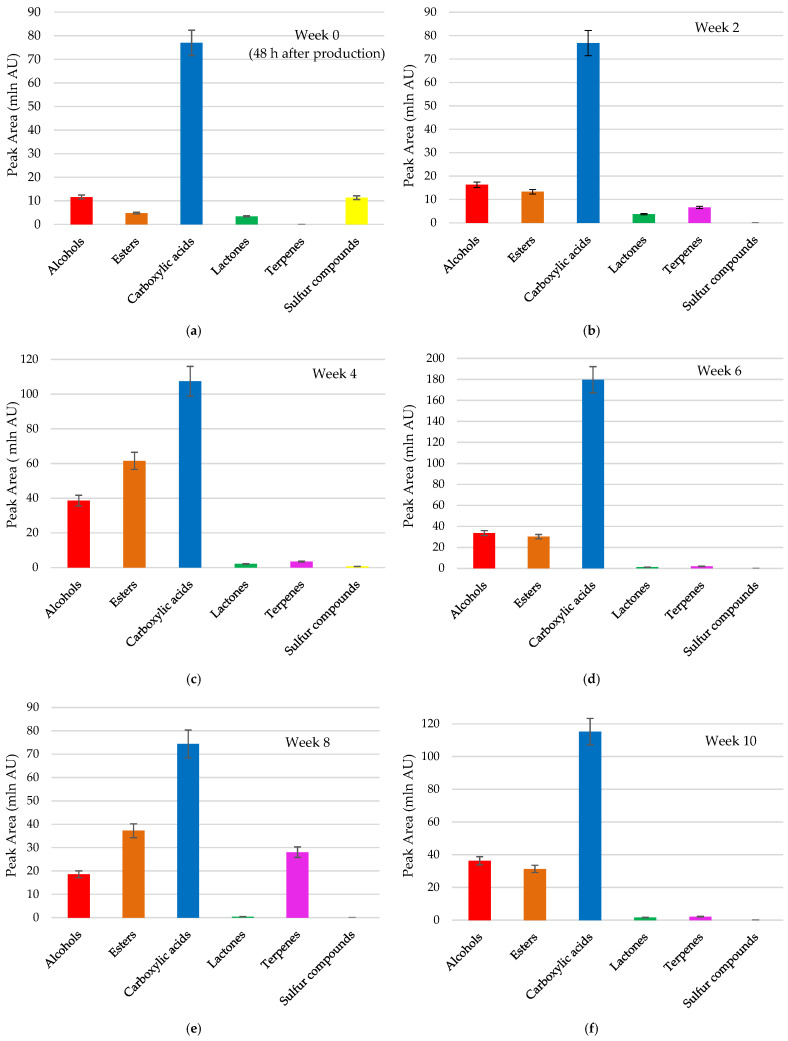
Distribution of volatile organic compound (VOC) classes at Week 0 (**a**), Week 2 (**b**), Week 4 (**c**), Week 6 (**d**), Week 8 (**e**), and Week 10 (**f**) of the cheese ripening process.

The maturation of the cheese matrix can be viewed as a biological relay, where the metabolic footprint of indigenous microflora drives the sequential evolution of the VOC profile (Figure 4a–f). This progression is initiated by the rapid growth of native lactic acid cocci, whose early dominance establishes the essential acidic and sulfur-based foundation of the young curd. The transition from primary acidification to complex aroma synthesis is triggered by the mass autolysis of these cocci between Weeks 2 and 4. This biological event serves as a critical turning point. The release of intracellular enzymes into the matrix acts as a catalyst for the sharp rise in ester concentrations observed in the subsequent stages. A secondary metabolic wave emerges around Week 6, coinciding with the proliferation of more resilient lactic acid rods. Their peak abundance directly correlates with the maximum accumulation of carboxylic acids, which define the final sensory profile. The eventual stabilization of the VOC profile in the final weeks (8–10) indicates that the native microflora has reached a state of technological equilibrium, primarily due to the natural exhaustion of available substrates and the accumulation of metabolic byproducts.

### 3.3. Dynamics of VOC Profiles During Maturation

Figure 2 and Figure 4a–f illustrate the shifting concentrations of primary VOC chemical classes throughout the cheese ripening process. This data reveals unique developmental patterns across various compound categories, reflecting the chemical progression toward technological maturity.

#### 3.3.1. Dynamics and Metabolic Evolution of Volatile Alcohols

The biochemical formation of volatile alcohols in artisanal raw cow-milk cheese is a multifaceted process driven by the primary and secondary metabolism of the indigenous microflora. According to Banks et al. [33], these compounds in non-pasteurized cheeses originate from three principal metabolic routes: the fermentation of residual lactose and citrates yielding short-chain alcohols, the catabolism of amino acids via the Ehrlich pathway, and the lipolysis of milk triglycerides, which serves as the primary source of glycerol.

The group of volatile alcohol compounds exhibits a highly dynamic and non-linear trend throughout the 10-week maturation period (Figure 2). From the onset of the process at Week 0 (Figure 4a) to the first major metabolic peak in Week 4 (Figure 4c), the total alcohol abundance surged by over 215%. This initial intensification is strictly correlated with the population dynamics of the indigenous LAB. The rapid decline in the lactic cocci count during the early maturation phase (Figure 1) facilitated the mass autolysis of these cells and the subsequent release of intracellular enzymes. As established by Ferrara et al. [38], early bacterial autolysis represents a critical catalytic bottleneck for flavor acceleration in artisanal dairy products, as the liberated enzymes catalyze the synthesis of alcohols and esters.

Following this initial surge, a sharp reduction of approximately 50% in total alcohol abundance was observed by Week 8 (Figure 2), indicating their utilization as essential precursors for esterification reactions. During these reactions, alcohols react with free fatty acids to produce fruity esters that define the piquant character of aged cheese. The ripening process ended with a secondary acceleration in alcohol synthesis between Week 8 and Week 10, characterized by an increase of nearly 95%, signifying the achievement of technological maturity in the cheese (Figure 4f).

**Figure 5:**
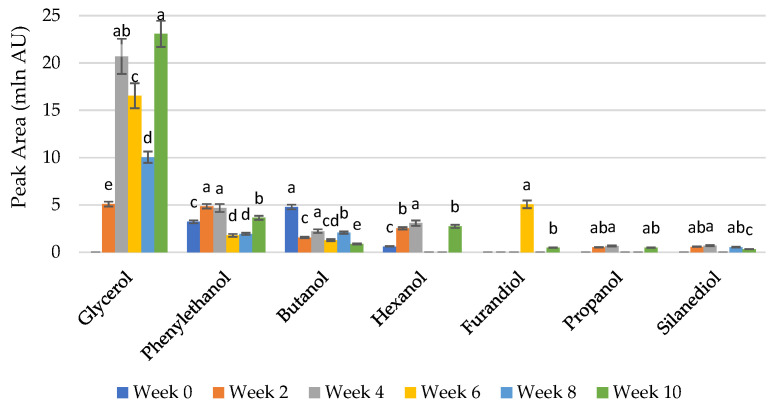
Distribution of individual volatile alcohols during the 10-week cheese maturation process. Values marked with different letters are significantly different (*p* < 0.05) and concern changes in the peak area of a specific compound during the time of ripening.

The qualitative profile of alcohol is dominated by seven primary compounds, with glycerol and phenylethanol acting as the most significant markers. Their individual profile reveals distinct metabolic shifts within the maturing matrix (Figure 5).

Phenylethanol reached its maximum concentration early in the maturation process at Week 2. According to Banks et al. [33], phenylethanol is synthesized through the transamination and subsequent reduction of phenylalanine, a process highly active during the initial stages of ripening. As noted by Curioni et al. [39], phenylethanol contributes delicate floral and rose-like notes, forming a foundation for the aromatic complexity and the final bouquet of the matured product.

Glycerol exhibited the most significant increase within the study, expanding by over 340% between Week 2 and Week 10 (Figure 5). This late-stage accumulation aligned with the dominance of rod-shaped NSLAB, which reached its maximum population density at Week 6. McSweeney et al. [40,41] suggest that the proliferation of polyols like glycerol contributes to the body and perceived smoothness of the cheese matrix, effectively balancing the sharpness of accumulated carboxylic acids.

The divergence between these markers highlights the transition from primary fermentation to a phase of sensory refinement. While butanol concentration plummeted by over 80% as early as Week 2, other compounds such as furandiol displayed highly specific peaks at Week 6 and then nearly disappeared at Week 10 (Figure 5). The complex and non-linear evolution of volatile alcohols confirms that this fraction functions as a central hub for flavor development rather than a static repository of fermentation byproducts. As stated by Atasever et al. [18], the sustained metabolic activity of the indigenous microflora, even during the natural growth inhibition phase toward the end of ripening, ensures the final accumulation of alcohols that characterize technological maturity. The transition from an alcoholic base generated by cocci to the refined polyol-rich matrix driven by rod-shaped NSLAB ultimately defines the unique volatile identity of artisanal raw-milk cheese.

#### 3.3.2. Dynamics and Metabolic Evolution of the Esters

The ester fraction represents one of the most dynamic components of the volatile profile during the 10-week ripening period of cheese, characterized by an unprecedented metabolic intensification followed by a stabilization phase (Figure 4a–f). In the complex biochemical ecosystem of raw cow-milk cheese, the formation of volatile esters occurs primarily through two enzymatic pathways: esterification and alcoholysis. While esterification involves the condensation of alcohols with free fatty acids, alcoholysis—the transfer of an acyl group from acyl-glycerols or acyl-CoA to an alcohol molecule—is considered the dominant route in many cheese varieties. In artisanal maturation, these reactions are fundamentally driven by the diverse enzymatic spectrum of the indigenous microflora.

At the onset of maturation (Week 0), esters were present only in trace amounts, reflecting the initial biochemical state of the curd (Figure 4a). However, as ripening progressed, a massive intensification occurred. Between Week 0 and Week 4, the total abundance of esters increased by approximately 900%, reaching the absolute maximum noted during the study (Figure 4c). This pulse-like change serves as a biochemical marker of the transition from fresh curd to a matured dairy product, where the initial baseline traces are rapidly replaced by aromatic molecules. According to Ferrara et al. [38], such rapid fluctuations in VOC profiles are definitive markers of microbial succession and can be utilized to predict the optimal ripening duration in artisanal cheeses.

The unique surge in esters at Week 4 (Figure 2) is inextricably linked to the population dynamics of the indigenous lactic acid bacteria. This quantitative peak correlates precisely with the autolysis phase of indigenous lactic acid cocci. As the cocci population declined sharply from its peak at Week 2 toward its autolysis phase by Week 4, the resulting cell lysis released a high concentration of intracellular esterases into the cheese matrix (Figure 1).

These enzymes provided the necessary catalytic environment to transform primary fermentation products into high-value aromatic compounds. It is crucial to clarify that the esterification process is mechanistically dependent on the cell lysis described in Section 3.1. The esterases responsible for the synthesis of volatile esters are primarily intracellular. Therefore, their release into the cheese matrix following the autolysis of the lactic acid cocci population is the prerequisite step that facilitates the subsequent, rapid intensification of ester formation observed at Week 4.

**Figure 6:**
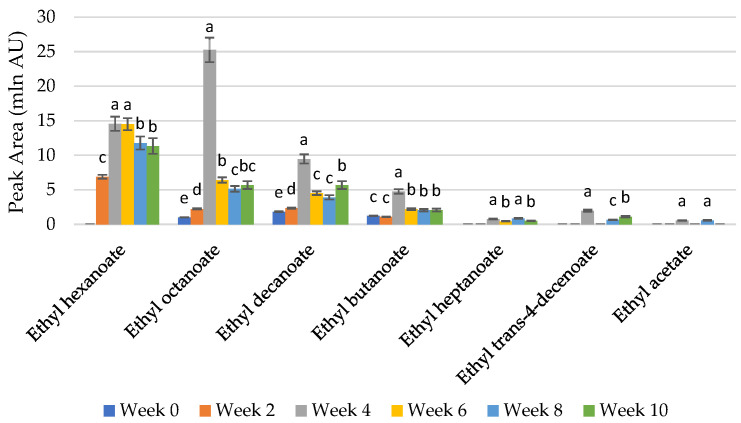
Distribution of individual volatile esters during the 10-week cheese maturation process. Values marked with different letters are significantly different (*p* < 0.05) and concern changes in the peak area of a specific compound during the time of ripening.

The individual ester profile is characterized by the dominance of ethyl esters, with ethyl hexanoate and ethyl octanoate emerging as the primary contributors to the aromatic foundation of the cheese (Figure 6). Akbulut Çakır [42] identifies these specific esters as critical components of the sensory identity of traditionally ripened varieties. Ethyl octanoate displayed the most aggressive growth changes, with its concentration increasing by 1000% between Week 2 and Week 4, highlighting the specific preference of the released esterases for medium-chain fatty acid substrates.

Ethyl hexanoate reached its maximum concentration at Week 4 but demonstrated a significantly more sustained presence throughout the maturation process. Its concentration doubled between Week 2 and Week 4 and persisted as a stable aromatic marker until the end of the 10-week study, showing a post-peak decline of only 15% (Figure 6). According to Pérez-Martín et al. [43] and Liu et al. [44], the sensory contribution of the ester fraction is important, acting as a primary counterweight to the intense acidic background. Esters impart fruity, sweet, and floral nuances, such as notes of pineapple and apricot, which serve to mask or balance the aggressive, pungent, and soapy attributes characteristic of accumulated short-chain and medium-chain fatty acids.

Following the Week 4 peak, a significant quantitative reduction was observed, with total ester levels decreasing by nearly 65% by Week 6 (Figure 4d). This stabilization phase coincides with the rise in NSLAB rods, which attained maximum population density while the primary cocci diminished. Although these rods contribute to a high concentration of precursor carboxylic acids, their metabolic activity favors the maintenance of an aromatic equilibrium rather than a rapid increase in esterification.

As noted by Pisana et al. [45], the sustained presence of fruity esters like ethyl hexanoate is vital for ensuring a complex and stable sensory profile as the product reaches technological maturity. The final ester profile represents a balance between the enzymatic heritage of the early cocci and the metabolic regulation provided by the late-stage lactic acid rods, marking the successful transformation of the raw milk into a refined artisanal product.

#### 3.3.3. Dominance and Metabolic Evolution of Carboxylic Acids

Carboxylic acids represent the most abundant class of VOCs identified throughout the 10-week ripening process, consistently maintaining the highest peak area (Figure 4a–f). The total abundance of this class exhibits a steady and significant upward trend from the initial stage through the mid-ripening phase, reflecting a complex biochemical mechanism primarily driven by the lipolytic and glycolytic activities of the indigenous microflora. According to Thierry et al. [46] and McSweeney et al. [40], these metabolic pathways are the fundamental drivers of carboxylic acid synthesis in non-pasteurized dairy products.

From Week 0 to the global metabolic peak at Week 6, the total concentration of carboxylic acids increased by approximately 125% (Figure 2 and Figure 4d). Such a significant change in the volatile profile aligns with the observations of Maslov Bandić [47] and Mureşan et al. [36], who describe carboxylic acids as the primary chemical markers for assessing the quality and maturity of artisanal dairy products. Following this maximum, a characteristic decline of roughly 55% was observed by Week 8, before a final stabilization occurred toward the end of the study at Week 10 (Figure 4f). This stabilization is essential to prevent excessive rancidity while fostering a complex and refined organoleptic distinctiveness, as noted by Dárgere et al. [48].

**Figure 7:**
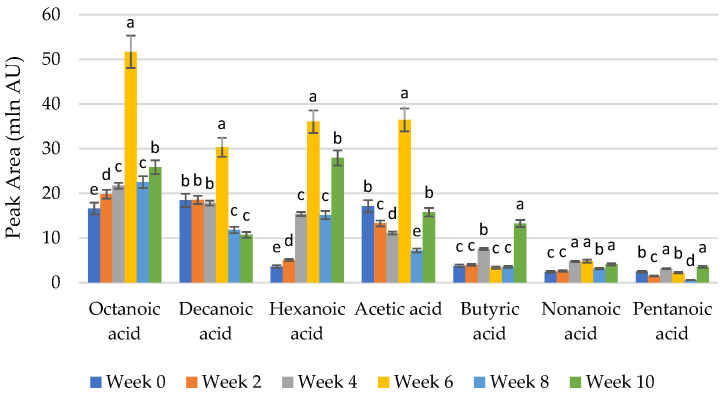
Distribution of individual volatile carboxylic acids during the 10-week cheese maturation process. Values marked with different letters are significantly different (*p* < 0.05) and concern changes in the peak area of a specific compound during the time of ripening.

The individual acid profile is dominated by medium-chain fatty acids (MCFAs), which are primary products of lipolysis, alongside short-chain fatty acids (SCFAs) derived from carbohydrate fermentation (Figure 7).

Octanoic acid is the most prominent compound in this class, which showed a consistent rise, peaking at Week 6 with an increase of nearly 210% compared to the Week 0 level. Despite a decline in the subsequent weeks, it remained the most abundant acid at Week 10, stabilized at a level 55% higher than the initial production stage (Figure 7). Hexanoic acid exhibited the most rapid increase in changes among the acids. Between Week 0 and Week 6, its presence increased by approximately 750% (an 8.5-fold change). It maintained a high concentration through the end of ripening, ending with a 560% increase (Figure 7). Acetic acid remained relatively stable during the first month but underwent a massive metabolic increase at Week 6: it tripled in concentration compared to the Week 4 level. Decanoic acid reached its peak at Week 6 after a 65% increase from Week 4 and then showed a unique trend with a sharp decline of 60% in the final stages, suggesting its potential conversion into other aromatic precursors like esters or lactones. Butyric acid was present in lower quantities, yet exhibited a late-stage intensification by increasing over 200% between Week 8 and Week 10, thus contributing to the piquant sensory notes of the mature cheese.

The evolution of the carboxylic acid profile serves as a direct chemical reflection of the life cycle and metabolic vigor of the indigenous microflora (Figure 1). The initial presence of acids at Week 0 (Figure 4a) is attributed to the primary fermentation conducted by indigenous lactic acid cocci. However, the massive accumulation observed between Week 4 and Week 6, where total acids more than doubled, is perfectly synchronized with the proliferation of rod-shaped non-starter lactic acid bacteria (NSLAB). As the rod population reached its absolute maximum density of 8.6 log CFU/g at Week 6, their intense lipolytic and proteolytic activities drove the peak concentrations of hexanoic, octanoic, and acetic acids (Figure 7). Research by Ferrara et al. [38] confirms that tracking such metabolic pulses is essential for understanding the transition from raw milk to a stabilized, matured matrix. The subsequent inhibition of growth and the decline in LAB populations after Week 6 directly correlate with the stabilization of the acid profile, which prevents excessive rancidity while ensuring the depth of the matured cheese’s flavor.

The analysis of carboxylic acids confirms their role as the quantitative basis of the volatile profile in raw milk cheese. The data demonstrate that while cocci initiate the acidic foundation, the rod-shaped NSLAB are the primary architects of the acid peak at Week 6. The successful transformation of raw milk components into this complex acidic matrix, as observed by Pisana et al. [45], signifies the achievement of full technological maturity of cheese.

#### 3.3.4. Dynamics and Metabolic Evolution of the Lactone Fraction

Lactones are specialized oxygenated heterocyclic compounds that serve as potent odorants, defining the creamy, fruity, and sweet depth of artisanal raw-milk cheese. Unlike other VOCs that may accumulate steadily, the lactone fraction exhibits a more irregular, successional chemistry during maturation. The metabolic origin of these compounds in non-pasteurized bovine systems is primarily initiated in the intramolecular cyclization of hydroxy fatty acids. These precursors are liberated from milk fat triglycerides through the specific lipase activity of the indigenous microbiota. Sensory analysis confirms that the transition from ƴ-decalactone to dodecalactone acts as a crucial aromatic fix. These molecules impart desirable buttery, coconut-like, and peach-like notes that provide a counterweight to the pungent acidic notes, ensuring a complex and evolving sensory profile that distinguishes raw-milk products from standardized industrial varieties [47].

Unlike carboxylic acids or alcohols, the total abundance of lactones followed a regressive trend as maturation progressed (Figure 4a–f). The highest total concentration was observed during the initial stages (Week 0 to Week 2), coinciding with the early dominance of the cocci microflora. Between Week 2 and Week 8, the total lactone fraction underwent a dramatic reduction of approximately 85% (Figure 4e), before showing a slight recovery in the final week of ripening.

**Figure 8:**
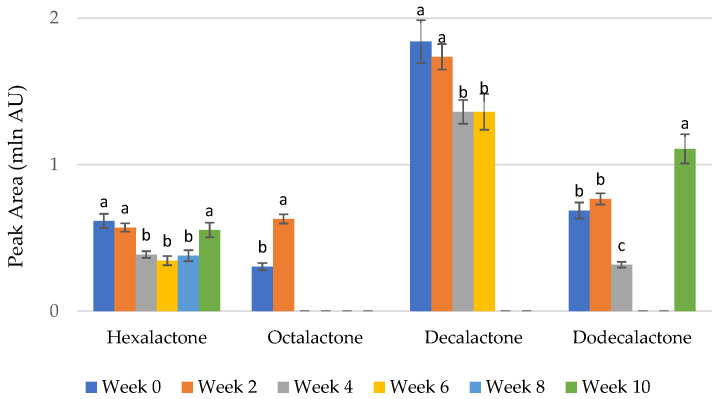
Distribution of individual volatile lactones during the 10-week cheese maturation process. Values marked with different letters are significantly different (*p* < 0.05) and concern changes in the peak area of a specific compound during the time of ripening.

The individual profile is characterized by four main compounds: hexalactone, octalactone, ƴ-decalactone, and dodecalactone, each exhibiting distinct temporal behaviors (Figure 8). Ƴ-decalactone emerged as the quantitatively dominant lactone during the first half of the ripening period. It maintained high stability between Week 0 and Week 2, but subsequently entered a steep decline (Figure 8). Exhibiting a specific window, octalactone reached its maximum abundance at Week 2, showing a 2-fold increase (+100%) compared to the initial stage of maturation. However, this was followed by a near-total depletion at Week 4, when its concentration had decreased by 94%, remaining at trace levels for the duration of the study (Figure 8). Dodecalactone displayed the unusual V-shaped metabolic trend. After an initial peak at Week 2, it decreased by 88% at Week 6. Remarkably, during the final stage of maturation (between Week 8 and Week 10), dodecalactone underwent a massive 12-fold (1200%) increase, becoming the most prominent lactone in the technologically mature cheese (Figure 8). In contrast to the heavier lactones, hexalactone remained the most stable component. It showed a gradual bi-weekly decline of approximately 10–15% through Week 6, followed by a 50% recovery in the final two weeks of ripening (Figure 8).

The dynamics of lactones appear to be intrinsically linked to the metabolic vigor and subsequent lysis of the indigenous LAB populations. The early peaks of ƴ-decalactone and octalactone (Weeks 0–2) are synchronized with the maximum proliferative phase of indigenous lactic acid cocci recorded during this interval (Figure 1). These bacteria likely facilitate the release of hydroxy acid precursors through lipase activity. The subsequent lack of these lactones between Week 4 and Week 8 strongly correlates with the autolysis phase of the cocci and the massive surge in carboxylic acids; it is hypothesized that these lactones may be undergoing ring-opening reactions or further chemical transformations as the pH and chemical environment change.

The unexpected late-stage surge in dodecalactone at Week 10 correlates with the prolonged presence of lactic acid rods. Although the rod population peaked at Week 6 and gradually declined thereafter, their sustained metabolic activity in the mature cheese matrix appears to drive the late-stage cyclization of hydroxy acids into dodecalactone. This suggests that while indigenous cocci establish the initial lactone profile, the rod-shaped lactic acid bacteria are responsible for the definitive aromatic stabilization of the cheese as it attains technological maturity.

The analysis reveals that lactones in raw milk cheese are highly transient metabolites. The transition from Week 2 to Week 4 marks a significant chemical turning point where early-stage lactones are rapidly depleted. The final status of the cheese at Week 10 is characterized by a lactone shift, where dodecalactone replaces ƴ-decalactone as the primary compound.

The 85% collapse of this fraction between the second and eighth weeks represents a critical metabolic point. As the environment becomes increasingly saturated with carboxylic acids, the early-stage lactones like octalactone likely submit to the changing pH levels. This phenomenon is characteristic of non-standardized artisanal systems where the acid surge can trigger ring-opening reactions, effectively scavenging these fruity precursors before they can stabilize. Recent findings by Bintsis et al. [49] suggest that such dynamic fluctuations in VOC fingerprints are essential for distinguishing raw milk products from those produced with commercial starters.

The most remarkable finding in the volatile lactone fraction is the V-shaped metabolic recovery of dodecalactone in the final stage of ripening. The 1200% increase compensates for the earlier loss of other volatiles. While the primary cocci have long since entered their autolysis phase, the sustained metabolic vigor of the lactic acid rods ensures that hydroxy acids are eventually cyclized into heavier, more stable lactone structures. The lactone shift from ƴ-decalactone to dodecalactone serves as a chemical signature of technological maturity [47,49,50].

#### 3.3.5. Dynamics and Metabolic Evolution of the Terpene Compounds

Terpene compounds in artisanal raw cow-milk cheese represent a unique category of VOCs, as they primarily originate from animals’ diet rather than microbial fermentation. These molecules are sequestered by the grazing animal and transferred into the milk fat. They remain entrapped within the complex fat and protein matrix during the early stages of curd formation. Their late-phase occurrence at the end of maturation is not a byproduct of synthesis, but a physical consequence of the deep structural reorganization of the cheese matrix. This liberation is triggered by the peak enzymatic activity of dominant NSLAB rods, whose lipolytic and proteolytic activity facilitates the biochemical changes in the matrix, effectively unlocking these markers. From a sensory perspective, the liberation of terpenes conveys delicate floral, balsamic, and herbaceous notes to the final product, contributing to its overall organoleptic characteristics. The transition toward a profile enriched with terpenes, such as ß-myrcene, indicates a multi-layered aroma of the cheese. This specific volatile signature acts as a reliable chemical marker for distinguishing long-matured, pasture-derived artisanal products from their standardized industrial counterparts [51].

The terpene fraction exhibits a distinct late-occurrence profile compared to other volatile classes, showing minimal presence in the early weeks followed by a massive increase in the final stages of maturation (Figure 4a–f). While total terpenes remained at trace levels during the first month, the profile underwent a significant intensification starting at Week 8. Between Week 6 and Week 8, the total abundance of terpenes increased by more than 8-fold (a 700% increase), reaching its maximum (Figure 4e). Although a general decline (by 40%) was observed in the final week of ripening, the terpene levels at Week 10 remained significantly higher than those recorded during the first six weeks (Figure 4f).

**Figure 9:**
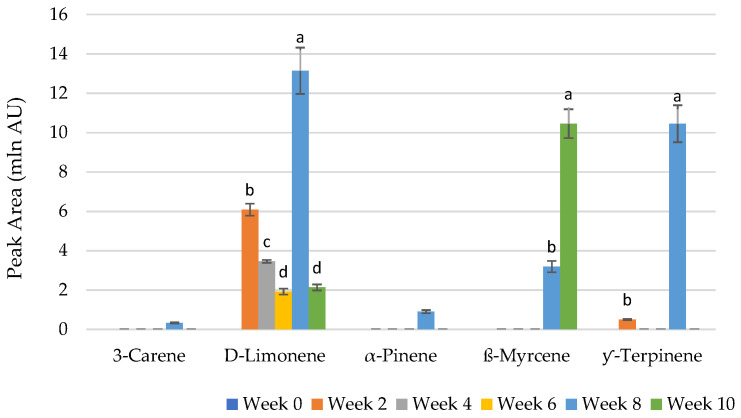
Distribution of individual volatile terpenes during the 10-week cheese maturation process. Values marked with different letters are significantly different (*p* < 0.05) and concern changes in the peak area of a specific compound during the time of ripening.

The individual profile is dominated by five primary compounds: D-Limonene, ß-Myrcene, ƴ-Terpinene, 3-Carene, and α-Pinene (Figure 9). D-Limonene remained the dominant terpene throughout the study. It showed an initial 4-fold increase between Week 0 and Week 2, followed by a gradual 65% decrease through Week 6 as it likely became re-trapped or degraded. A secondary and more powerful increase occurred at Week 8, where its concentration increased by over 6-fold (+550%) compared to the Week 6 level. However, it declined again by 80% at the end of the study (Figure 8). Ƴ-Terpinene exhibited a pulse behavior. It appeared in small quantities at Week 2, then remained undetectable until Week 8, at which point it showed a huge increase, becoming one of the most abundant terpenes in the profile (Figure 9). Its presence was short-lived as it declined by over 95% by Week 10. Displaying the most delayed release pattern, ß-Myrcene remained at trace levels until the very end of the ripening process. Between Week 8 and Week 10, it underwent a massive 3.5-fold increase (+250%), replacing D-Limonene and ƴ-Terpinene as the dominant terpene at the stage of technological maturity (Figure 9). The minor components 3-Carene and α-Pinene occurred only at Week 8, and both declined close to the end of the 10-week period (Figure 9).

The appearance of terpenes is strictly correlated with the stages of matrix degradation driven by the succession of indigenous LAB. The relative absence of terpenes during the cocci-dominated phase (Weeks 0–4) suggests that these compounds remained isolated within the complex fat and protein matrix of the raw milk curd. Even the autolysis of cocci at Week 4 did not trigger a significant release of these compounds (Figure 4c). The massive surge in Week 8 is an indirect result of the peak activity of lactic acid rods. As the rods reached their maximum population at Week 6, their intense lipolytic and proteolytic enzymes began a severe breakdown of the cheese structure. This biological softening of the matrix facilitated the liberation of sequestered dietary terpenes, leading to the quantitative peak observed at Week 8. The subsequent change at Week 10 (specifically the rise in ß-Myrcene) reflects the final stage of biochemical transformation as the rods’ population begins to decline, marking the achievement of a terpene-rich equilibrium. The terpene analysis demonstrates that these compounds serve as indicators of advanced ripening and matrix breakdown. The significant 8-fold increase at Week 8 acts as a chemical marker for the cumulative enzymatic impact of the indigenous NSLAB. The transition from a Limonene- to a Myrcene-dominant terpene profile at Week 10 shows that the raw milk cheese has achieved its full sensory complexity, liberated from the molecular constraints of the initial curd. The identification of significant quantitative pulses in these specific volatile markers provides a robust chemical framework for the authentication of traditionally ripened artisanal products. This analytical approach effectively distinguishes long-matured raw-milk cheeses from standardized industrial counterparts by characterizing the unique successional transformations driven by the native microbiota.

#### 3.3.6. Dynamics and Metabolic Evolution of Volatile Sulfur Compounds

The maturation of artisanal raw milk cheese is characterized by a distinct temporal accumulation-depletion track of volatile sulfur compounds, which serve as markers of early biochemical changes. Unlike other VOC classes that accumulate over time, sulfur compounds reach their absolute maximum 48 h after cheese production (Week 0). Following this initial peak, the total relative abundance of this class undergoes a drastic reduction of over 90% within the first two weeks (Figure 4b). For the rest of the 10-week period, volatile sulfur compounds remain at trace levels, indicating a rapid transformation or loss of these molecules (Figure 4c–f). Research by Gaya et al. [52] highlights that these volatile sulfur substances constitute a distinctive biochemical mark of raw milk matrices, wherein the autochthonous microbiota facilitates a significantly more expansive enzymatic set for amino acid catabolism than that provided by standardized commercial starter cultures.

**Figure 10:**
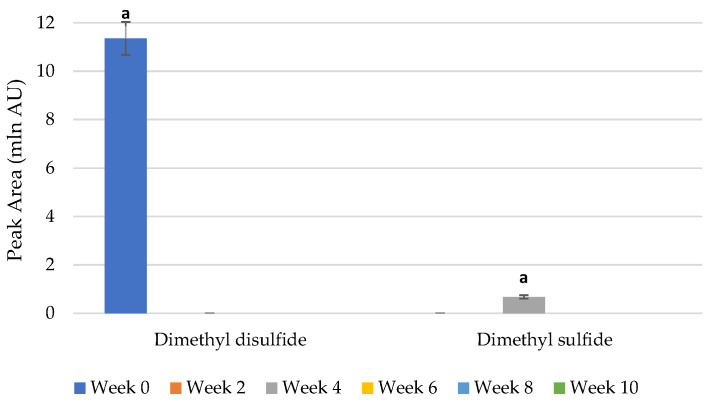
Distribution of individual volatile sulfur compounds during the 10-week cheese maturation process. Values marked with different letters are significantly different (*p* < 0.05) and concern changes in the peak area of a specific compound during the time of ripening.

The sulfur profile is primarily defined by two compounds: dimethyl disulfide and dimethyl sulfide, which show contrasting behaviors during the first month of ripening (Figure 10). Dimethyl disulfide is the primary contributor to the initial sulfur peak at Week 0. Its relative abundance is at its maximum during the first 48 h after production. However, as the cheese enters the second week of ripening, dimethyl disulfide levels undergo a massive 30-fold reduction (a 97% decrease), effectively disappearing from the detectable volatile profile for the rest of the study (Figure 10). In contrast, dimethyl sulfide is present in much lower relative abundance at Week 0. It exhibits a distinct late appearance at Week 4, where its peak area increases by approximately 3-fold (200%) compared to the initial production day (Figure 10). Following this brief intensification, it undergoes a total depletion, becoming undetectable from Week 6 onwards.

The dynamics of sulfur compounds are closely tied to the initial metabolic activity and subsequent succession of the raw milk microflora. The high relative abundance of dimethyl disulfide at Week 0 correlates with the maximum population of indigenous LAB cocci. At this early stage, the cocci are highly active in the young curd, driving the catabolism of sulfur-containing amino acids like methionine and cysteine. The significant 30-fold drop in this compound by Week 2 suggests that as the environment becomes more acidic and the cocci begin to stabilize, the pathways leading to disulfide formation are inhibited or the compounds are lost to the atmosphere due to their high volatility. The secondary occurrence of dimethyl sulfide at Week 4 (Figure 10) is synchronized with the autolysis phase of the cocci. As the cocci population declines, the release of intracellular enzymes into the matrix likely triggers a final, brief liberation of dimethyl sulfide from protein precursors. The total disappearance of volatile sulfur compounds by Week 6 coincides with the peak of the lactic acid rods. The rod-shaped microflora appears to favor metabolic pathways directed toward carboxylic acid production rather than sulfur metabolism. As elucidated by Esmaeilzadeh et al. [53] in their study of traditional Kurdish Kope cheese, the metabolic transition from sulfur compounds toward complex esters is fundamental to the sensory evolution and flavor stability of traditionally ripened varieties.

The analysis of sulfur compounds identifies them as markers of early-stage fermentation in raw milk cheese. The massive 97% reduction in dimethyl disulfide after Week 0 serves as a chemical indicator of the transition from the initial curd phase to the formal ripening process. The brief appearance of dimethyl sulfide at Week 4 provides a chemical footprint of cocci autolysis. By Week 10, the absence of these compounds confirms the change toward a more stable and complex aroma profile, characteristic of the long-term maturation driven by the indigenous rod-shaped microflora.

#### 3.3.7. Future Research Directions

Based on the foundational insights established in this study regarding the macro-kinetic successional dynamics of indigenous LAB and the volatile metabolome of ripening raw-milk cheese, several critical pathways emerge for future multi-omics investigation. A primary forward-looking trajectory involves the transition from standard culture-dependent enumeration to high-throughput, culture-independent molecular methodologies. Although the utilization of M17 and MRS agar provided essential, high-resolution quantitative data regarding the physiological fluxes of broad microbial cohorts (cocci versus rods), these classical phenotypic media inherently obscure profound inter- and intra-species genetic diversity within the mature matrix. Morphologically identical colonies frequently mask a multi-taxa consortium characterized by highly divergent enzymatic pathways. Consequently, resolving taxonomic identification at the precise species and strain levels remained beyond the analytical scope of this study. Employing Next-Generation Sequencing (NGS) platform configurations, such as 16S rRNA amplicon metagenomics, is fundamentally required to map the exact taxonomic architecture of these autochthonous architects. Dissecting these populations down to individual species boundaries—such as differentiating between *Lactococcus lactis* subspecies or pinpointing distinct non-starter *Lactobacillus*, *Lactiplantibacillus*, or *Limosilactobacillus* strains—is crucial, as volatile organic compound (VOC) biosynthesis profiles are strictly strain-specific attributes. Resolving this taxonomic gap will enable future multi-omics models to pair precise genomic code with the specific volatilomic surges characterized in this study.

Furthermore, to fully elucidate the enzymatic mechanisms behind the biosynthesis of volatile metabolites, an integrated multi-omics approach is recommended. Combining the current HS-SPME/GC-MS metabolomic profiling with metatranscriptomics could clarify the active metabolic pathways during critical phases, such as the autolysis-mediated release of esterases and the late-stage liberation of sequestered terpenes. Such research would provide definitive proof of the link between gene expression in the indigenous microflora and the resulting volatile fingerprint.

From a sensory perspective, future studies should incorporate gas chromatography-olfactometry (GC-O) and comprehensive quantitative sensory analysis. Determining the odor activity values (OAVs) of individual compounds would help identify the specific key odorants that define the unique organoleptic identity of raw-milk cheese.

Finally, expanding the research to include different milk sources (e.g., sheep’s milk) or geographical regions would strengthen the proposed molecular authentication model, ultimately supporting the standardization of quality control for traditional artisanal products on a broader scale.

#### 3.3.8. Study Limitations

While this study provides valuable mechanistic insights into the synchronized biological relay driving cheese ripening, a rigorous interpretation of the volatilomic and microbiological data requires a transparent acknowledgment of several structural and methodological boundary conditions. Primarily, regarding geographic and seasonal constraints, the experimental design was deliberately restricted to a single organic farm and a single winter milking season. While this specific setup successfully minimized external environmental noise and provided an excellent baseline for evaluating unadulterated indigenous microflora succession, it inherently limits the immediate generalization of the biological relay model across multi-regional dairy matrices or variations driven by seasonal pasture diets.

Beyond these geographic parameters, a critical taxonomic resolution boundary must be considered, as the microbial enumeration relied entirely on traditional culture-dependent phenotypic monitoring using selective M17 and MRS media. Although these classical protocols provided high-resolution quantitative kinetics of broad morpho-physiological cohorts, they lack the capacity to resolve strain-level taxonomy or unveil unculturable microbial fractions that might contribute minor volatile notes to the final bouquet. Concurrently, the profiling of volatile organic compounds (VOCs) via HS-SPME/GC-MS introduces an analytical boundary condition, given that it was conducted using relative peak area metrics derived from total ion chromatograms rather than absolute mass concentration quantification based on multi-point internal standard calibration curves. Consequently, the reported dynamics reflect relative compositional shifts rather than absolute chemical yields.

Finally, the investigation is bounded by its localized sample size footprint, operating with a matrix of 18 cheese wheels produced across two independent experimental batches. While extensive triplicate sampling at six distinct chronological steps ensured high technical reproducibility and strict statistical validation within this study, expanding the batch scale in subsequent trials remains necessary to further validate the model’s industrial robustness and widespread adaptability.

## 4. Conclusions

This investigation successfully validates the synchronized biological relay model as a grounded analytical framework for characterizing the volatile profiling of artisanal raw-milk cheese. The research demonstrates that flavor development is not a linear progression but a diverse, non-linear successional process driven by the milk’s indigenous microflora, where the chronological transition from active lactic cocci to dominant lactobacilli rods acts as an empirical indicator for specific volatile synthesis waves. The study confirms that the mass autolysis of native cocci between weeks 2 and 4 constitutes a key catalytic milestone, releasing intracellular enzymes into the matrix that facilitate a substantial intensification of the ester and alcohol fractions. The subsequent proliferation of resilient, rod-shaped non-starter lactic acid bacteria ensures the stabilization of the core carboxylic acid matrix, concluding in a state of technological equilibrium by Week 10, where transient early odorants are systematically replaced by a stable, refined matrix of esters, alcohols, and heavy lactones like dodecalactone.

On an analytical level, this study contributes to dairy microbiology by establishing a detailed empirical baseline that explicitly bridges the population dynamics of native microflora with the temporal evolution of the volatile metabolome. By mapping these specific metabolic shifts against the biological life cycles of unpasteurized milk populations, the manuscript offers a rigorous characterization of open-fermentation cheese systems within defined experimental boundaries. This integrated volatolomic mapping advances the understanding of non-starter microbial ecology, providing traditional dairy science with an analytical approach to evaluate product identity and quality attributes based on unadulterated native flora instead of commercial starter cultures.

Furthermore, the characterization of delayed, matrix-mediated volatile releases—such as the late-ripening liberation of sequestered dietary terpenes driven by advanced matrix reorganization at Week 8—offers a useful analytical checkpoint for assessing the typicity and authenticity of pasture-derived artisanal cheeses. While acknowledging the structural boundaries of a localized sample size, this integrated approach successfully demonstrates the profound role of native flora in establishing the complex volatile matrix of traditional dairy products. Ultimately, these findings provide a valuable scientific stepping stone, offering a balanced and transparent methodology to support quality characterization and product differentiation in the non-pasteurized cheese sector.

## Figures and Tables

**Table 1:** Chronological evolution of core physicochemical parameters during the 10-week ripening process of artisanal raw-milk cheese.

Ripening Period	pH	Water Activity (a_w_)	Moisture Content (%)	Salt Content (NaCl %)	Fat Content (%)
Week 0	5.26 ± 0.03	0.976 ± 0.002	44.21 ± 0.52	1.31 ± 0.06	28.45 ± 0.38
Week 2	5.14 ± 0.02	0.964 ± 0.003	40.85 ± 0.44	1.65 ± 0.05	30.12 ± 0.41
Week 4	5.02 ± 0.04	0.955 ± 0.002	38.12 ± 0.39	1.88 ± 0.04	31.95 ± 0.35
Week 6	5.06 ± 0.03	0.948 ± 0.002	36.44 ± 0.41	2.04 ± 0.07	33.10 ± 0.48
Week 8	5.15 ± 0.02	0.944 ± 0.003	35.18 ± 0.33	2.15 ± 0.05	34.05 ± 0.31
Week 10	5.22 ± 0.03	0.941 ± 0.001	34.25 ± 0.28	2.22 ± 0.04	34.82 ± 0.26

Values represent the mean ± standard deviation of triplicate analyses. Fat content is reported on a total weight basis.

## Data Availability

The original contributions presented in this study are included in the article. Further inquiries can be directed to the corresponding authors.

## Abbreviations

The following abbreviations are used in this manuscript:

LAB	Lactic Acid Bacteria
VOCs	Volatile Organic Compounds
HS-SPME/GC-MS	Headspace Solid-Phase Microextraction/Gas Chromatography–Mass Spectrometry
NSLAB	Non-Starter Lactic Acid Bacteria
CFU/g	Colony-Forming Units per gram
TIC	Total Ion Chromatogram
AU	Arbitrary Units
SD	Standard Deviation
ANOVA	Analysis of Variance
PCA	Principal Component Analysis
